# Histone deacetylase 6 activity is critical for the metastasis of Burkitt’s lymphoma cells

**DOI:** 10.1186/s12935-014-0139-z

**Published:** 2014-12-05

**Authors:** Ning Ding, Lingyan Ping, Lixia Feng, Xiaohui Zheng, Yuqin Song, Jun Zhu

**Affiliations:** Key laboratory of Carcinogenesis and Translational Research (Ministry of Education), Department of Lymphoma, Peking University Cancer Hospital & Institute, No.52 Fucheng Road, Haidian District, Beijing, 100142 China

**Keywords:** Burkitt’s lymphoma, histone deacetylase 6, Cell shape elongation, Metastasis, Microtubule dynamics

## Abstract

**Background:**

Burkitt’s lymphoma is an aggressive malignancy with high risk of metastasis to extranodal sites, such as bone marrow and central nervous system. The prognosis of metastatic Burkitt’s lymphoma is poor. Here we sought to identify a role of histone deacetylase 6 (HDAC6) in the metastasis of Burkitt’s lymphoma cells.

**Methods:**

Burkitt’s lymphoma cells were pharmacologically treated with niltubacin, tubacin or sodium butyrate (NaB) or transfected with siRNAs to knock down the expression of HDAC6. Cell migration and invasion ability were measured by transwell assay, and cell cycle progression was analyzed by flow cytometry. Cell adhesion and proliferation was determined by CellTiter-Glo luminescent cell viability assay kit. Cell morphological alteration and microtubule stability were analyzed by immunofluorescence staining. Effect of niltubacin, tubacin and NaB on acetylated tubulin and siRNA efficacy were measured by western blotting.

**Results:**

Suppression of histone deacetylase 6 activity significantly compromised the migration and invasion of Burkitt’s lymphoma cells, without affecting cell proliferation and cell cycle progression. Mechanistic study revealed that HDAC6 modulated chemokine induced cell shape elongation and cell adhesion probably through its action on microtubule dynamics.

**Conclusions:**

We identified a critical role of HDAC6 in the metastasis of Burkitt’s lymphoma cells, suggesting that pharmacological inhibition of HDAC6 could be a promising strategy for the management of metastatic Burkitt’s lymphoma.

## Background

Burkitt’s lymphoma (BL) as a highly aggressive B-cell malignancy, usually occurs in adolescent as well as in patients with immune defect. Endemic BL is the most common variant and prevails in Africa where almost all the patients are found with Epstein-Barr Virus (EBV) infection [1,2]. Besides, there are two other BL variants: sporadic BL which accounts for about 30-50% of childhood lymphomas in the developed countries, and HIV infection caused immune-deficient associated type [3]. BL grows rapidly, potentially doubling in size every day, which leads to its sensitivity to chemotherapeutic agents. Currently most of the childhood BL is effectively managed with the cyclical intensive chemotherapy [4]. However, another feature of BL is its high aggression, occasionally disseminates to bone marrow (BM) and central nervous system (CNS), contributing to poor prognosis in clinics [5]. Therefore, attempts to explore better regimens to inhibit the metastasis of BL is urgently needed.

Histone deacetylases (HDACs) are a superfamily comprising of 18 proteins, which regulate gene expression through deacetylation of histones to produce a highly compact chromatin structure [6,7]. Besides, HDACs interact with many non-histone substrates to regulate diverse cellular activities, including cell division, cell motility, and angiogenesis [8,9], making targeting HDACs being a promising approach for treatment of various malignancy. Several HDAC inhibitors have demonstrated excellent inhibitory effects on tumor growth [10], for instance, panobinostat, a pan-HDAC inhibitor, hold great promise in several hematological malignancy including cutaneous T-cell lymphoma, Hodgkin lymphoma, and B-cell lymphoma in both preclinical study and clinical trials [11]. However, due to the significance of HDACs in cellular activities, severe adverse effects, such as thrombocytopenia are also observed. Therefore, elucidating the role of each HDAC member in tumors could shed light to the development of better regimens against cancers. HDAC6 is a unique member of HDAC family, which is localized predominantly in the cytoplasm [12]. Unlike the other HDAC members, HDAC6 bears two catalytic HDAC domains and has minimal effect on cell cycle related gene expression and cell proliferation [13], making its role in malignant tumors elusive. In this study, we adopted tubacin, niltubacin (deacetylase inactive tubacin derivatives), and sodium butyrate (NaB) to elucidate the role of HDAC6 in BL. Tubacin is a specific inhibitor of HDAC6, while NaB is a HDAC activity which lacks activity on HDAC6 [12]. Our data demonstrated that inhibition of HDAC6 activity significantly suppressed SDF-1α induced cell shape elongation and cell adhesion, thereby leading to impaired cell motility without affecting cell proliferation.

## Results

**Figure 1:**
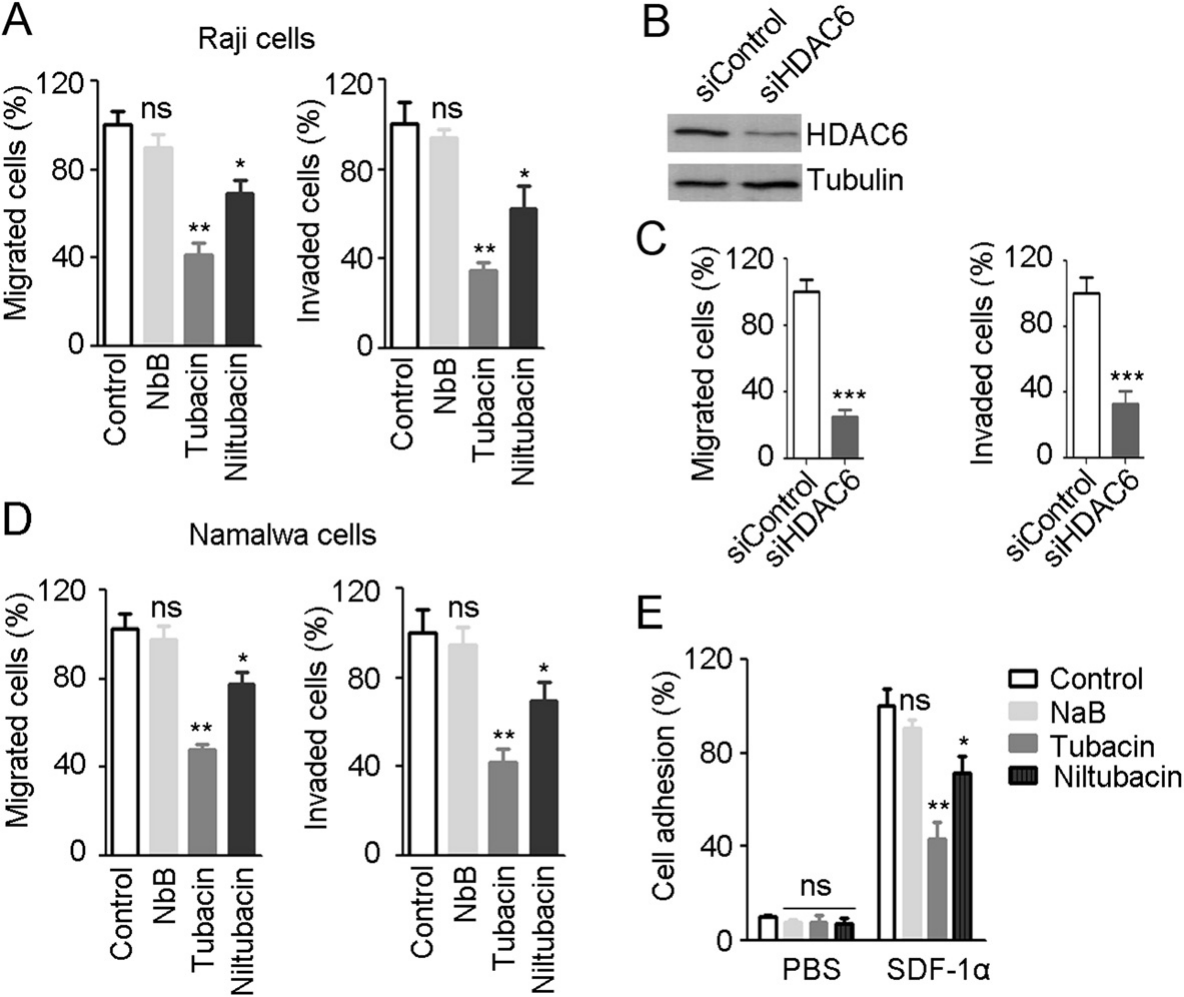
**Inhibition of HDAC6 significantly compromises the motility and adhesion of BL cells. (A)** Raji cells cells pretreated with DMSO, NaB, tubacin or niltubacin were seeded into the inserts which were coated without or with Fibronectin. 12 hour later, cells in the lower chambers were analyzed by FCAS. **(B)** Raji cells were transfected with siRNAs for 48 hours, and the knockdown efficacy was determined by western blotting. **(C)** Raji cells transfected with siRNAs were seeded into transwell inserts for migration and invasion detection. **(D)** Migration and invasion analysis of Namalwa cells treated with DMSO, NaB, tubacin or niltubacin. **(E)** Raji cells treated with DMSO, NaB, tubacin or niltubacin were stimulated with SDF-1α for 3 hours, and then cells were seeded to the wells which were precoated with Fibronectin. Cells were allowed to adhere for 30 minutes, and adherent cells were measured by CellTiter-Glo luminescent cell viability assay kit. *, **, *** indicate P <0.05, 0.01, 0.001 versus DMSO, respectively; ns, not significant.

Firstly we investigated the role of HDAC6 in BL cell motility. Raji cells cells were plated into the inserts that were precoated with or without Fibronectin for invasion and migration study, respectively. Invaded or migrated cells were collected 12 hours later and analyzed by FACS. As shown in Figure 1A, tubacin and niltubacin treatment remarkably compromised SDF-1α induced motility of BL cells, whereas DMSO or NaB exposure had no obvious effect on cell motility. To confirm the observation we knocked down the expression of HDAC6 by using siRNA (Figure 1B), and found that siHDAC6 treatment markedly decreased the migration and invasion of Raji cells (Figure 1C). To examine the generalization role of HDAC6 in BL cell motility, we assayed on another BL Namalwa cells. Consistently, similar results were observed in Namalwa cells (Figure 1D). As cell attachment to endothelium or extracellular matrix (ECM) is prerequisite for malignant cell infiltration to the destined sites, we examined whether HDAC6 regulates cell motility via its action on cell adhesion. SDF-1α stimulation significantly enhanced the cell adhesion to Fibronectin, whereas tubacin or niltubacin exposure markedly compromised SDF-1α induced cell adhesion (Figure 1E). By contrast, NaB treatment showed little reduction of cell adhesion (Figure 1E). Taken together, these findings reveal the HDAC6 is critical for SDF-1α induced cell motility and cell adhesion.

**Figure 2:**
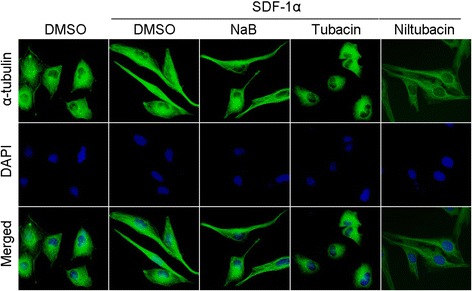
**The role of HDAC6 in SDF-1α induced cell shape elongation.** Raji cells pretreated with DMSO, NaB,tubacin or niltubacin for 3 hours were stimulated with SDF-1α, and then cells were immunostained with anti-α-tubulin (green) antibody and DAPI (blue).

Cell motility is a complex process which is exquisitely orchestrated and involves a multiple of proteins which collaborate with cytoskeletons to facilitate directed movement. The first step is the formation of polarized cells towards the movement direction, accompanied with cell shape elongation and asymmetry distribution of cellular molecules. To test whether HDAC6 regulates lymphoma cell motility through affecting cell shape elongation, Raji cells pretreated with DMSO, NaB, tubacin, or niltubacin were stimulated with SDF-1α to induce cell movement. Interestingly, cells became elongated in the presence of SDF-1α (Figure 2A, left panels). By contrast, compared with NaB or niltubacin treatment, tubacin exposure remarkably inhibited the SDF-1α induced cell shape elongation (Figure 2A, right panels). Collectively, these data suggest that HDAC6 activity is essential for chemokine induced cell shape elongation, which is critical for cell polarity and motility.

**Figure 3:**
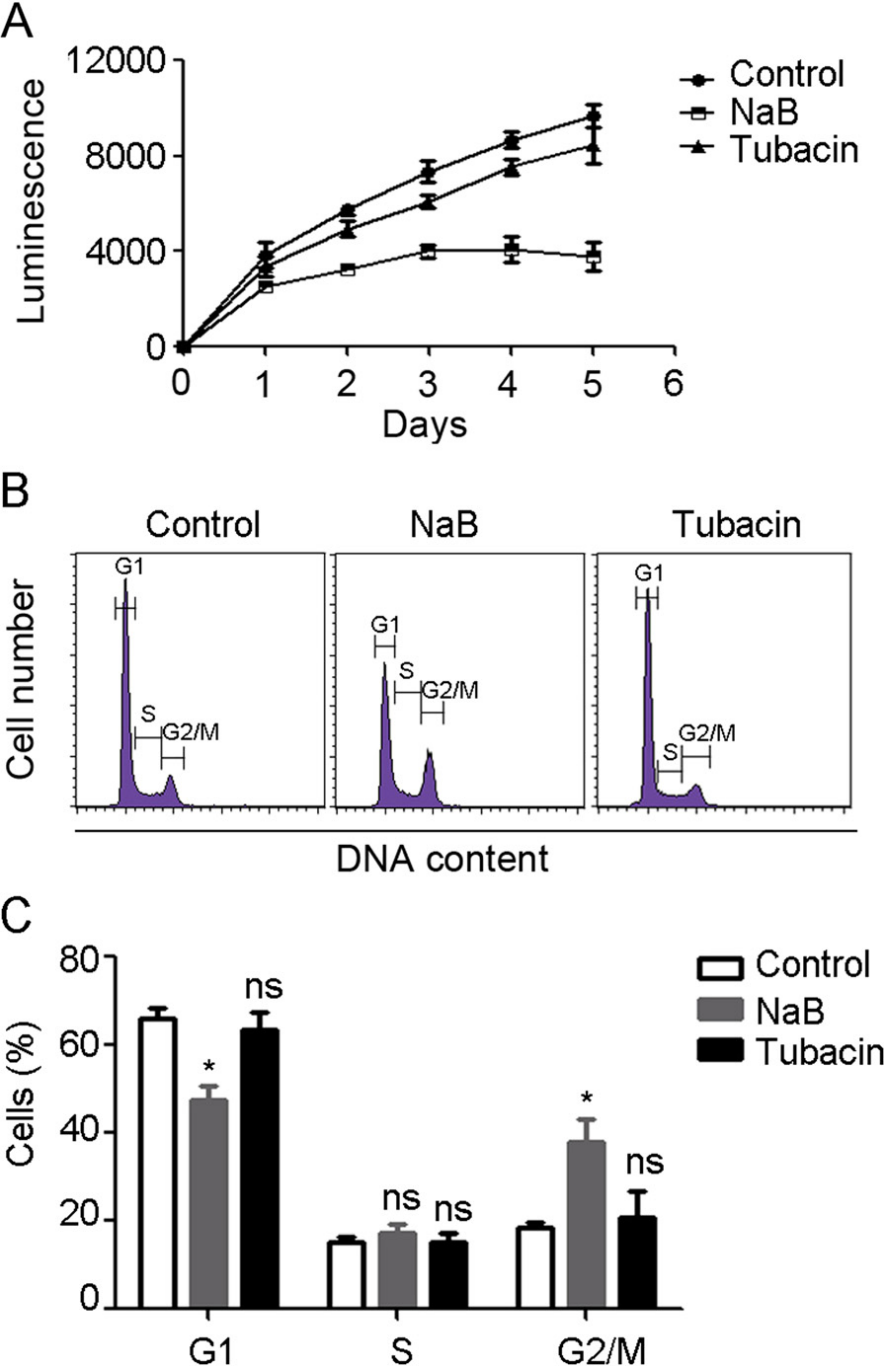
**HDAC6 does not affect the cell proliferation and cell cycle progression. (A)** Raji cells were treated with DMSO, NaB, or tubacin for the indicated time, and cell viability was determined by CellTiter-Glo luminescent cell viability assay. **(B)** Cell cycle analysis of Raji cells treated with DMSO, NaB, or tubacin. **(C)** Experiments were performed as in panel B, and the percentage of cells in G1, S, and G2/M phases was calculated. *, P < 0.05 versus control; ns, not significant.

HDAC inhibitors dramatically shrink several types of cancers in clinical trials. We thus investigated the involvement of HDAC6 in lymphoma cell growth. Raji cells were treated with DMSO, NaB, or tubacin for the indicated time and cell viability was measured. As shown in Figure 3A, NaB treatment significantly suppressed the proliferation of BL Raji cells, however, little cytotoxicity was observed in the tubacin treatment group. To confirm the results observed in cell proliferation, we analyzed the effect of HDAC6 on cell cycle progression. Raji cells were stained with PI for DNA content analysis. NaB treatment decreased the percentage of cells at G1 phase and arrested cells at G2/M phase, whereas DMSO or tubacin treatment did not affect the percentage of cells at G1, S, and G2/M phases compared with that of the NaB treatment group (Figure 3B). Collectively, these data reveal that HDAC6 activity is independent from HDAC-mediated cell proliferation and cell cycle progression.

**Figure 4:**
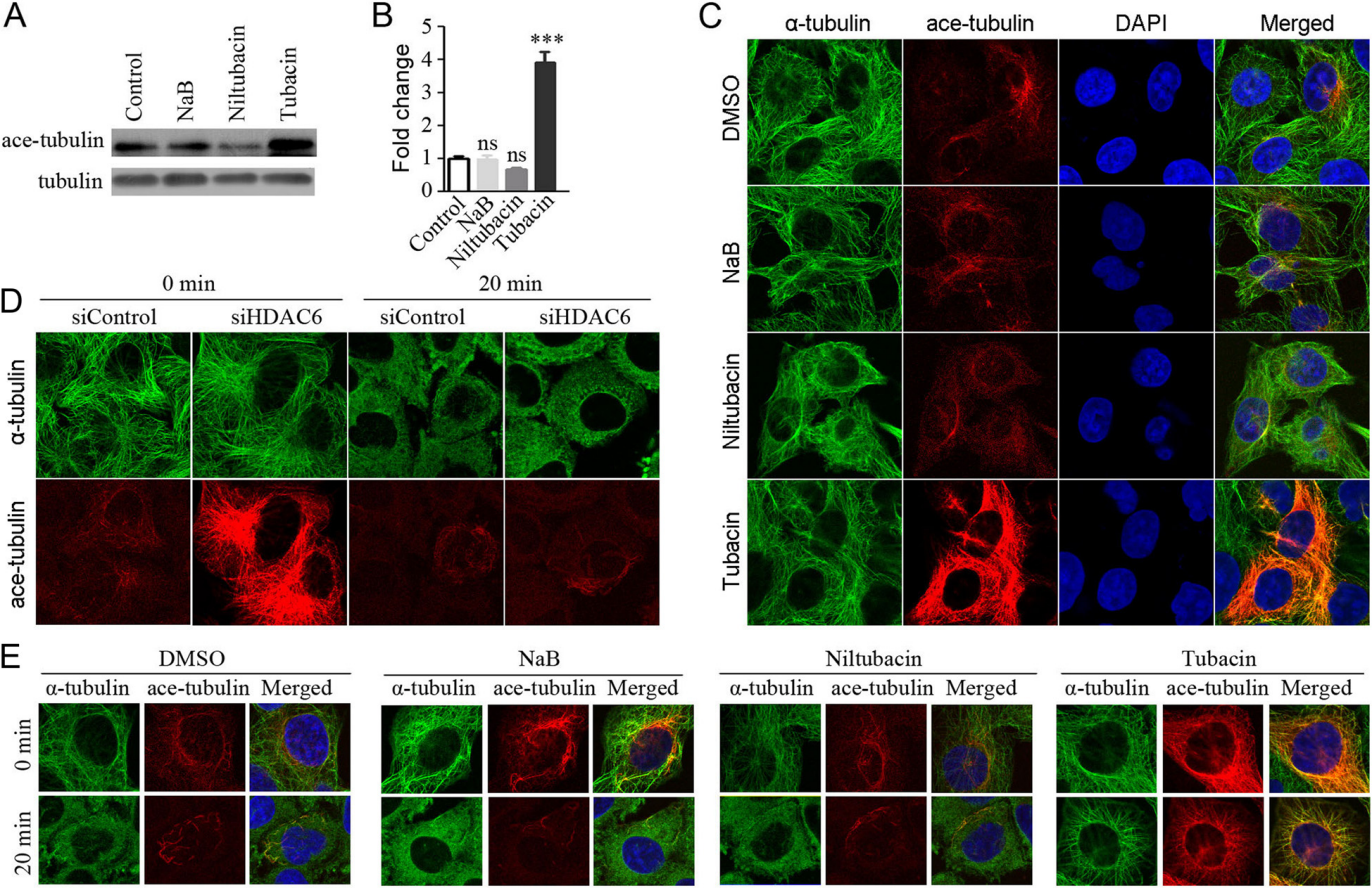
**Suppression of HDAC6 markedly enhances the acetylation of microtubules. (A)** Raji cells treated with DMSO, NaB, niltubacin, or tubacin were lyzed and the acetylated tubulin was determined by western blotting. **(B)** Experiments were performed as in panel A, and relative ratio of acetylated tubulin to total tubulin was determined. **(C)** Raji cells treated with DMSO, NaB, niltubacin or tubacin were immunostained with anti-α-tubulin (green), anti-acetylated tubulin (red) antibodies and DAPI (blue). **(D)** HDAC6-depleted Raji cells were incubated on ice for 20 minutes to depolymerize microtubules and then cells were fixed and immunostained with antibodies against α-tubulin (green) and acetylated tubulin (red). **(E)** Raji cells pretreated with DMSO, NaB, niltubacin or tubacin for 24 hours were incubated on ice for 20 minutes and then cells were immunostained with antibodies against α-tubulin (green) and acetylated tubulin (red), and DAPI (blue). ***, P <0.001 versus DMSO; ns, not significant.

As microtubules are the key component regulator of cell motility, we then examined whether HDAC6 regulates Raji cell elongation, motility, and adhesion through its action on microtubule dynamics. As shown in Figure 4A and B, tubacin exposure remarkably elevated the level of acetylated microtubules, whereas NaB or niltubacin treatment did not increase the microtubule acetylation. By immunostaining of acetylated microtubules we confirmed the observation in immunoblotting, tubacin treatment significantly contributed to enhanced microtubule acetylation in cells (Figure 4C). To elucidate whether the regulation of microtubule dynamics is dependent on the tubulin deacetylase activity or HDAC6 itself binding to the microtubules, we tested the microtubule stability of HDAC6-depleted cells in cold-induced depolymerizing condition. Depletion of HDAC6 did not promote the microtubule stability (Figure 4D), whereas pharmacological inhibition of HDAC6 with tubacin resulted in an increase in microtubule stability against cold-induced microtubule disassembly (Figure 4E). To sum up, these data reveal an important role of HDAC6 in chemokine induced cell shape elongation and cell motility, and implicates that HDAC6-mediated microtubule dynamics might be the underlying mechanism of cell shape elongation and motility.

## Discussion

BL is an aggressive B cell malignant disease with high risk of metastasis to extranodal sites. The prognosis for BL with involvement of BM and CNS is poor. Thus, attempts to explore agents to inhibit the metastasis of BL is greatly needed. In this study, we found that HDAC6 plays a crucial role in SDF-1α induced cell morphological changes. It is noteworthy that cell polarization towards elongated shape is prerequisite for cell migration and invasion [14,15]. Further study confirmed that suppression of HDAC6 significantly restrained the motility of Raji cells. These findings thus suggest that targeting HDAC6 might be a potential strategy for management of metastatic BL.

HDAC inhibitors have been proved to be effective for several types of cancer, immune disorders, and neurodegenerative diseases [16-18]. Currently almost all the extensively studied HDAC inhibitors are non-selective, such as vorinostat, romidepsin, and panobinostat [10]. In spite of excellent potency on shrinking tumors, these agents lead to inevitable adverse effects sometimes. Thus, elucidating the exact mediator responsible for the inhibitory effect on tumors is obligated. It is reported that HDAC2 suppresses the expression of p53, causing cell survival and insensitivity to chemotherapeutic agents [19,20]. In addition, loss of HDAC5 restrains the cell proliferation of hepatocellular carcinoma by regulation of p21 and cyclin D1, thereby leading to cell cycle arrest and apoptosis [21]. In this scenario, we revealed that HDAC6 did not affect the proliferation and cell cycle progression of Raji cells, which is consistent with the results found in mouse embryonic stem cells [13]. Regarding little cytotoxicity of HDAC6 inhibitors in BL cells, it would be interesting in the future to investigate the efficiency of HDAC6 inhibitors combined with genotoxic agents or radiotherapy for the management of advanced BL.

It is believed that microtubule cytoskeleton is exquisitely modulated to fulfill their distinct function, such as mitosis, differentiation, directed cell movement, and vehicle transport [22-24]. HDAC6 is identified as a microtubule-binding protein, which regulates microtubule dynamics through deacetylation of α-tubulin [12]. In addition, several other HDAC6 substrates have been identified, including cortactin, MAP7 domain-containing protein 3 (Mdp3), and heat shock protein 90 (HSP-90) [25-27]. As microtubule dynamics is essential for the establishment of cell polarity, turnover of focal adhesion, and cell motility, we concentrated on α-tubulin in this study. Our data revealed that microtubule stability against cold-induced depolymerizing condition was promoted in pharmacologically treated cells other than in HDAC6-depleted cells, which was consistent with the observation found in MCF-7 cells [28]. Taken together, these findings demonstrate that HDAC6 modulates the acetylation level of α-tubulin, leading to chemokine induced microtubule remodeling and directed movement of BL cells, which provides the basis for pharmacological inhibition of HDAC6 as a potential approach for metastatic BL.

## Materials and methods

### Materials

Tubacin (HDAC6 selective inhibitor) and NaB (a HDAC inhibitor without activity on HDAC6) were purchased from Santa Cruz Biotech (CA, USA). Niltubacin (deacetylase inactive tubacin derivatives) was purchased from Abcam (MA, USA). Antibodies against α-tubulin and acetylated tubulin were purchased from Abcam (MA, USA). SDF-1α, DAPI and propidium iodide (PI) were form Sigma (MO, USA), and CellTiter-Glo luminescent cell viability assay kit was from Promega (WI, USA). Fibronectin and Transwell were purchased from BD Biosciences (NJ, USA) and Corning (MA, USA), respectively. SiRNAs targeting luciferase (control) and HDAC6 were synthesized by Invitrogen (Beijing, China).

### Cell culture and transfection

Human Burkitt’s lymphoma Raji and Namalwa cells were purchased from the American Type Culture Collection (ATCC), and maintained in the RPMI1640 medium supplemented with 10% FBS. SiRNAs were transfected into cells by using lipofectamine 2000 (Invitrogen) according to the manufacturer’s instruction.

### Cell proliferation assay

Cells were seeded at 6000 cells/well 12 hours prior to DMSO, NaB, or tubacin exposure. At the indicated time, cell viability was determined by CellTiter-Glo luminescent cell viability assay kit according to the manufacture’s protocol.

### Immunoblotting

Cells were lyzed in cell lysis buffer containing 1% Triton X-100, 150 mM NaCl, and 50 mM Tris. Proteins were separated by 10% SDS-PAGE gel electrophoresis, and then transferred onto PVDF membranes. The membranes were blocked in Tris-buffered saline containing 5% fat-free dry milk and 0.2% Tween 20. After incubation with primary antibodies, the membranes were incubated with horseradish peroxidase-conjugated secondary antibodies. The proteins of interest were detected with enhanced chemiluminescence detection reagent according to the manufacturer’s instructions.

### Cell cycle progression analysis

Raji cells treated with DMSO, NaB (1 μM), or tubacin (1 μM) were collected and washed twice with ice-cold phosphate-buffered saline (PBS). Then cells were fixed with 70% ethanol at 4°C for 24 hours. After twice wash with PBS, cells were incubated with propidium iodide (PI)/RNase A solution for 30 minutes. Samples were examined with a BD FACS Calibur Flow Cytometer.

### Cell motility assays

Cell motility assays were carried out as described [29] with minor modification. For cell migration assay, Raji cells or Namalwa cells suspended in serum free medium were plated into inserts, and for cell invasion assay, cells suspended in serum free medium were plated into inserts which were precoated with Fibronectin (50 ng/ml) for overnight. DMSO, NaB (1 μM), or tubacin (1 μM) was added to the inserts, and then the inserts were placed in a 24-well plate containing the serum free medium supplemented with SDF-1α (100 ng/ml). 12 hours later, cells in the lower chambers were analyzed by a BD FACS Calibur Flow Cytometer.

### Cell adhesion assay

Prior to SDF-1α (100 ng/ml) stimulation, Raji cells were treated with DMSO, NaB (1 μM), or tubacin (1 μM) for 3 hours. Then cells were plated into a 96-well plate which were precoated with Fibronectin (50 ng/ml) to allow cell adherence for 30 minutes. Subsequently cells were washed with PBS three times to remove the non-adherent cells, and adherent cells were measured by CellTiter-Glo luminescent cell viability assay kit.

### Immunofluorescence microscopy

Immunofluorescence staining was performed as described [30]. In brief, cells grown on glass coverslips were fixed with 4% paraformaldehyde for 30 minutes. Cells were washed for three times with PBS, followed by incubation with 2% bovine serum albumin for 20 minutes. Then cells were sequentially incubated with primary antibodies and fluorescein-conjugated secondary antibodies. The nuclei were counterstained with DAPI. Coverslips were mounted with 90% glycerol in PBS and imaged with a Zeiss fluorescence microscope.

### Microtubule stability analysis

Raji cells were incubated with DMSO, NaB, niltubacin or tubacin or transfected with HDAC6 siRNA for 24 hours, then cells were incubated on ice for 0 or 20 minutes. Cells were then fixed and immunostained with antibodies against α-tubulin and acetylated tubulin.

### Statistical analysis

All data were derived from three independent experiments, and expressed as means ± SD. Student’s t-test and one-way analysis of variance (ANOVA) were performed for statistical analysis. P value < 0.05 indicates statistical significance.
